# Dosimetry-guided peptide receptor radionuclide therapy in neuroendocrine tumors: interim safety analysis of the DUONEN trial

**DOI:** 10.3389/fendo.2025.1716247

**Published:** 2025-12-01

**Authors:** Maciej Kolodziej, Marta Opalinska, Renata Mikolajczak, Alicja Hubalewska-Dydejczyk, Marek Dedecjus, Aldona Kowalska, Marek Saracyn, Piotr Garnuszek, Izabela Cieszykowska, Joanna Januszkiewicz-Caulier, Joanna Dlugosinska, Adam Daniel Durma, Katarzyna Jozwik-Plebanek, Adrianna Mroz, Katarzyna Janiak, Danuta Gasior-Perczak, Malgorzata Trofimiuk-Muldner, Anna Sowa-Staszczak, Janusz Braziewicz, Wioletta Lenda-Tracz, Krzysztof Kacperski, Anna Budzynska, Agata Kubik, Patrycja Pastusiak, Wioletta Chalewska, Anna Borkowska, Paulina Cegla, Agata Walecka-Mazur, Artur Szczodry, Grzegorz Kaminski

**Affiliations:** 1 Department of Endocrinology and Isotope Therapy, Military Institute of Medicine - National Research Institute, Warsaw, Poland; 2 Chair and Department of Endocrinology, Jagiellonian University Medical College, Krakow, Poland; 3 Radioisotope Centre POLATOM, National Centre for Nuclear Research, Otwock, Poland; 4 Department of Endocrine Oncology and Nuclear Medicine, National Institute of Oncology - National Research Institute, Warsaw, Poland; 5 Collegium Medicum, Jan Kochanowski University, Kielce, Poland; 6 Faculty of Medicine, University of Warsaw, Warsaw, Poland; 7 Department of Nuclear Medicine, Holy Cross Cancer Center, Kielce, Poland; 8 Faculty of Health Sciences, Jagiellonian University Medical College, Krakow, Poland; 9 Particle Acceleration Physics and Technology Division, National Centre for Nuclear Research, Otwock, Poland; 10 Department of Nuclear Medicine, Military Institute of Medicine - National Research Institute, Warsaw, Poland; 11 Department of Endocrinology, Holy Cross Cancer Center, Kielce, Poland

**Keywords:** NET, RLT, PRRT, dosimetry, tandem therapy, [^177^Lu]Lu-DOTA-TATE, [^90^Y]Y-DOTA-TATE, GEP-NET

## Abstract

**Background:**

PRRT with [^177^Lu]Lu-DOTA-TATE improves survival in advanced GEP-NETs, but fixed-activity dosing may result in undertreatment or unnecessary toxicity. Individualized dosimetry and tandem-PRRT with ^90^Y/^177^Lu have been proposed, but prospective randomized evidence is lacking.

**Methods:**

DUONEN is an ongoing multicenter, randomized phase 3 trial (N = 92 planned; 56 analyzed) comparing standard fixed-activity [^177^Lu]Lu-DOTA-TATE (arm A) with three dosimetry-guided regimens: arm B (^177^Lu+^90^Y, variable ^90^Y); arm C (^177^Lu+^90^Y, variable ^177^Lu); arm D (variable ^177^Lu). Organ dosimetry was performed after each cycle, with per-cycle activity modifications to respect kidney (23 Gy) and marrow (2 Gy) thresholds. Safety was assessed by laboratory, renal, and hepatic parameters.

**Results:**

Activity reductions predominated in arms B and C, while increases were common in arm D. Median cumulative kidney and marrow doses were highest in arm C (29.1 Gy and 0.79 Gy, respectively), driven by ^90^Y contribution. Hematologic declines were observed across all arms, most prominently in lymphocytes and platelets, and correlated with marrow dose but not with categorical dose modifications. Renal function remained stable, and no clinically relevant hepatotoxicity occurred.

**Conclusions:**

This interim analysis demonstrates the feasibility and safety of dosimetry-guided PRRT strategies, including individualized ^177^Lu escalation and tandem ^90^Y/^177^Lu. DUONEN provides the first randomized prospective evidence for isotope- and patient-tailored PRRT dosing. Long-term follow-up will clarify their impact on efficacy.

**Clinical trial registration:**

https://www.clinicaltrialsregister.eu/ctr-search/search?query=eudract_number:2020-006068-99, identifier 2020-006068-99.

## 1 Introduction

Although gastroenteropancreatic neuroendocrine tumors (GEP-NETs) are still considered rare, numerous reports and epidemiological studies in recent years have demonstrated a substantial increase in their incidence and detection (1, 2). In most cases, GEP-NETs are characterized by an indolent, long-term clinical course. A hallmark of this group of tumors is the overexpression of somatostatin receptors on the cell surface, which enables the use of long-acting somatostatin analogs (SSA) as first-line therapy (3, 4). According to both Polish and European guidelines, patients who experience disease progression during SSA therapy (and are not eligible for surgical treatment) are recommended to receive peptide receptor radionuclide therapy (PRRT, also referred to as radioligand therapy, RLT) as a second-line treatment (5–8). The NETTER-1 trial demonstrated the superiority of [^177^Lu]Lu-DOTA-TATE over standard therapy with respect to disease control and improvement in quality of life, which led to the approval of this therapy in the United States and Europe (9, 10).

Due to the heterogeneity of metastatic disease typically observed in GEP-NETs (coexistence of bulky lesions and small metastases), standard PRRT with [^177^Lu]Lu-DOTA-TATE may show limited effectiveness in some patients. Furthermore, there is evidence that a fixed regimen of four cycles of [^177^Lu]Lu-DOTA-TATE administered to all patients, without accounting for individual variability in radiosensitivity, may lead to undertreatment (11). This highlights the need for continuous development of PRRT methods and the search for optimal strategies regarding activity dosing and isotope selection. Retrospective studies have explored the combination of somatostatin analogs labeled with isotopes of different physical properties—such as lutetium-177 (^177^Lu), which has a maximum β^-^ particle tissue penetration range of approximately 2 mm, and yttrium-90 (^90^Y), with a range of 10–12 mm. This approach, referred to as “tandem-PRRT,” may theoretically improve tumor coverage across lesions of varying size (12–16). In the era of personalized medicine, prospective studies are warranted to compare different dosing strategies and the use of alternative isotopes in the treatment of GEP-NETs, as well as to evaluate the toxicity profiles of such approaches. Currently, no randomized trials have investigated the impact of tandem-PRRT on overall survival in GEP-NET patients, nor compared this method with conventional therapy based solely on [^177^Lu]Lu-DOTA-TATE. Importantly, the available evidence from non-randomized studies and clinical experience suggests a potential advantage of combining isotopes over monoisotope therapy.

DUONEN is the first Polish, randomized, multicenter clinical trial comparing different PRRT regimens. The aim of the study is to optimize treatment of GEP-NET patients by improving therapeutic efficacy while minimizing adverse events, particularly in critical organs such as bone marrow and kidneys. The novelty of DUONEN lies in a prospective, randomized, multicenter comparison of fixed-activity [^177^Lu]Lu-DOTA-TATE versus multiple dosimetry-guided PRRT strategies (including tandem-PRRT), with per-cycle adaptation. It is the first study to test several dosimetry algorithms head-to-head while quantifying isotope-specific contributions to organ doses.

## 2 Materials and methods

The DUONEN study (“The Use of Tandem LutaPol/ItraPol Therapy (^177^Lu/^90^Y-DOTATATE) as an Effective Approach in the Treatment of Neuroendocrine Tumors”) is ongoing, multicenter, open-label, phase 3 trial conducted at four clinical centers in Poland. The study was funded by the Medical Research Agency, Poland (project number 2019/ABM/01/00077). All procedures performed in this study involving human participants were conducted in accordance with the ethical standards of the institutional and/or national research committee and with the 1964 Declaration of Helsinki and its later amendments. The protocol was reviewed and approved by the Bioethics Committee of the Jagiellonian University, Kraków, Poland (approval no. 1072.61201.24.2020, dated January 20, 2021). Written informed consent was obtained from all individual participants included in the study. The study was registered in EudraCT 2020-006068-99 (September 20, 2021).

The protocol-defined sample size was 92 patients. Recruitment began in 2022. This pre-specified interim analysis, representing one of the study milestones, includes 56 patients (approximately 60% of the planned cohort) who completed PRRT according to their randomized assignment. PRRT administration in the last enrolled patient is expected to be completed in September 2027, with the final follow-up visit for the last patient scheduled for September 2032.

### 2.1 Baseline characteristics

A total of 56 DUONEN trial patients (**Table 1**) were included in the interim analysis, with 16 assigned to arm A (standard RLT with [^177^Lu]Lu-DOTA-TATE), 16 to arm B, 12 to arm C, and 12 to arm D (dosimetry-modified arms). The median age at enrollment was 69 years (range: 27–86), and 46.4% of patients were male. The majority of patients presented with gastroenteropancreatic NETs originating from the midgut (62.5%) and pancreas (28.6%), with smaller proportions from the hindgut, stomach, or other primary sites. Most tumors were well-differentiated G2 GEP-NETs (60.7%), followed by G1 (39.3%). At the baseline, all patients demonstrated somatostatin receptor–positive disease on imaging.

**Table 1:** Characteristics of DUONEN trial patients enrolled in the interim analysis.

Characteristic	Total (N = 56)	Arm A (N = 16)	Arm B (N = 16)	Arm C (N = 12)	Arm D (N = 12)
Median age, years (range)	69 (27-86)	68 (54-81)	73 (49-86)	69 (50-81)	62 (27-74)
Sex, n (%)					
male	26 (46.4%)	5 (31.3%)	5 (31.3%)	7 (58.3%)	9 (75.0%)
female	43 (53.6%)	11 (68.7%)	11 (68.7%)	5 (41.7%)	3 (25.0%)
Ethnicity, n (%)					
Caucasian	56 (100%)	16 (100%)	16 (100%)	12 (100%)	12 (100%)
Black	0 (0%)	0 (0%)	0 (0)%	0 (0%)	0 (0%)
Asian	0 (0%)	0 (0%)	0 (0%)	0 (0%)	0 (0%)
Median BMI, kg/m^2^ (range)	25.9 (17.7 - 36.6)	24.1 (20.1 - 30.1)	23.9 (19.9 - 33.3)	25.3 (17.7 - 33.2)	24.9 (20.2 - 36.6)
Primary site, n (%)					
Midgut	35 (62.5%)	10 (62.5%)	11 (68.8%)	7 (58.3%)	7 (58.3%)
Pancreas	16 (28.6%)	5 (31.5%)	4 (25.0%)	2 (16.7%)	5 (41.7%)
Stomach	1 (1.8%)	0 (0%)	1 (6.3%)	0 (0%)	0 (0%)
Hindgut	3 (5.4%)	0 (0%)	0 (0%)	3 (25.0%)	0 (0%)
Other/unknown	1 (1.8%)	1 (6.3%)	0 (0%)	0 (0%)	0 (0%)
Histological grade, n (%)					
NET G1	22 (39.3%)	8 (50.0%)	6 (37.5%)	2 (16.7%)	6 (50.0%)
NET G2	34 (60.7%)	8 (50.0%)	10 (62.5%)	10 (83.3%)	6 (50.0%)
Sites of metastases, n (%)					
Liver	52 (92.9%)	15 (93.8%)	14 (87.5%)	12 (100%)	11 (91.7%)
Lymph nodes	33 (58,9%)	8 (50.0%)	9 (56.3%)	7 (58.3%)	6 (50.0%)
Bone	22 (39.3%)	5 (31.3%)	6 (37.5%)	6 (50.0%)	5 (41.7%)
Other	37 (60.1%)	9 (56.3%)	11 (68.8%)	10 (83.3%)	7 (58.3%)
Median time from diagnosis, months (range)	69 (12–125)	69 (12–101)	71 (20–89)	66 (18–91)	70 (15–125)
Karnofsky performance status ≥80, n (%)	51 (91.1%)	14 (87.5%)	14 (87.5%)	12 (100.0%)	11 (91.7%)
Baseline labs (median, range)					
Hemoglobin, g/dL	12.9 (10.0 -16.2)	12.5 (10.0 -15.0)	13.3 (11.9 -15.7)	12.7 (10.6 -14.0)	13.1 (11.3 -16.2)
Platelets, ×10^9^/L	229 (148 –381)	266 (148 -377	248 (141 - 371	183 (168 - 296)	219 (162 - 343)
WBC, ×10^9^/L	6.62 (3.73 –10.81)	5.95 (3.89 - 10.04)	6.98 (5.74 - 10.93)	5.37 (5.08 - 10.51)	7.18 (3.73 - 10.81)
eGFR, mL/min/1.73 m²	82 (48 – 153)	81 (60 - 118)	76 (49 - 110)	81 (53 - 102)	82 (48 - 153)

Across the four study arms, the baseline demographic and disease characteristics were broadly comparable, with no clinically significant imbalances (**Table 1**).

Eligible patients were adults with histologically confirmed, disseminated or inoperable, well-differentiated gastroenteropancreatic neuroendocrine tumors (GEP-NETs), defined by a Ki-67 index of ≤20%. The main inclusion criteria included documented disease progression, as assessed by the Response Evaluation Criteria in Solid Tumors (RECIST), version 1.1, on computed tomography (CT) or magnetic resonance imaging (MRI) within a maximum of 18 months while receiving long-acting somatostatin analog therapy (octreotide LAR 30 mg or lanreotide 120 mg administered every 4 weeks).

Additional eligibility requirements included a good general health status, defined as a Karnofsky performance status of ≥60 and a life expectancy of >26 weeks, as well as positive somatostatin receptor expression in all target lesions. Patients were also required to have adequate organ function, defined as: serum creatinine <120 μmol/L or estimated glomerular filtration rate (eGFR) >45 mL/min/1.73 m²; hemoglobin >9.0 g/dL; white blood cell count >3 × 10^9^/L; and platelet count >100 × 10^9^/L within 4 weeks prior to enrollment. Liver function tests were required to be <3 times the upper limit of normal.

Key exclusion criteria included pregnancy or breastfeeding; a history of other malignancies; and any prior treatment with liver-directed transarterial therapy (e.g., embolization), chemotherapy, mTOR inhibitors, or PRRT at any time prior to randomization.

### 2.2 Trial design

Patients who met all the inclusion and did not meet any of the exclusion criteria were randomly assigned to one of four treatment arms:

- Arm A: [^177^Lu]Lu-DOTA-TATE administered at a fixed activity of 7.4 GBq per cycle.
- Arm B: A combination of [^177^Lu]Lu-DOTA-TATE and [^90^Y]Y-DOTA-TATE, initially in a ratio of 3.7:1.85 GBq/GBq. The activity of [^177^Lu]Lu-DOTA-TATE remained constant across all cycles, whereas the activity of [^90^Y]Y-DOTA-TATE was adjusted in the second, third, and fourth cycles based on bone marrow and kidney dosimetry to maximize radiation dose delivery to tumor tissue.
- Arm C: Analogous to Arm B, except that [^90^Y]Y-DOTA-TATE activity was kept constant, and the dose of [^177^Lu]Lu-DOTA-TATE was modified according to dosimetry findings.
- Arm D: [^177^Lu]Lu-DOTA-TATE administered at activity of 7.4 GBq at the first cycle, followed by individualized adjustment in subsequent cycles based on bone marrow and kidney dosimetry to maximize radiation dose delivery to tumor tissue.

All patients received four cycles of PRRT administered every 8 ± 2 weeks, unless exclusion criteria were met during the treatment course. Supportive care with long-acting somatostatin analogs was continued throughout: these were administered approximately 2 weeks after each PRRT cycle and then monthly following completion of therapy.

All patients received treatment in an inpatient setting within the Departments of Nuclear Medicine. Long-acting somatostatin analogs were discontinued 6 weeks prior to the initiation of PRRT and reintroduced 2 weeks after each treatment cycle.

Antiemetic prophylaxis consisted of ondansetron administered 30 minutes prior to the start of the amino acid infusion. An amino acid solution (1 L of Vamin 18, containing 18 g of lysine and 22.6 g of arginine in 2 L of solution) was initiated 60 minutes before PRRT administration and infused over 4 hours.

The PRRT infusion was administered intravenously over approximately 30 minutes.

On day 2 following PRRT, an additional 0.5 L infusion of Vamin 18 was administered following premedication with ondansetron.

Treatment discontinuation criteria during PRRT included any of the following: Karnofsky performance status score <60; serum creatinine >150 μmol/L; eGFR <30 mL/min/1.73 m²; hemoglobin <8.0 g/dL; white blood cell count <3 × 10^9^/L; neutrophil count <1 × 10^9^/L; platelet count <80 × 10^9^/L; or liver function test results exceeding 3 times the upper limit of normal. Patients meeting any of these criteria were withdrawn from further PRRT and transitioned to follow-up care.

### 2.3 Dosimetry of critical organs and PRRT dose calculations

For each patient, full dosimetry of critical organs and selected GEP-NET lesions was performed following each cycle of PRRT.

Bone marrow dosimetry was based on measurements of isotope activity in peripheral blood, as well as in hot organs (liver, spleen, kidneys) and the rest of body, estimated from the four SPECT images listed below, to account for the cross-dose to red marrow. Blood samples were collected at five time points: 5 minutes, 10 minutes, 40 minutes, 4 hours (immediately prior to the first SPECT/CT scan), and 24 hours after each RLT administration.

Renal dosimetry was performed using 4 post-therapeutic SPECT/CT scans obtained at 4, 24, 48, and 192 hours after PRRT administration. Data were analyzed using dedicated Q-DOSE software. Kidney absorbed dose calculations were performed using the IDAC 2.1 model. In select anatomical variants, such as a horseshoe kidney, the VOXEL S model was applied instead.

Activity of ^177^Lu was directly quantified using post-therapeutic imaging, while ^90^Y activity was estimated based on ^177^Lu-derived images, under the assumption of identical biodistribution for both isotopes.

Dose constraints for critical organs were defined based on previously published data:

- Kidneys: a cumulative absorbed dose limit of 23 Gy for the entire PRRT course.
- Bone marrow: a maximum of 0.5 Gy per cycle, with a total dose not exceeding 2 Gy across all cycles.

### 2.4 PRRT safety evaluation

Safety in all PRRT arms is assessed based on the incidence and severity of adverse events, graded according to the Common Terminology Criteria for Adverse Events (CTCAE), version 5.0. Safety evaluations include:

- Laboratory assessments, including complete blood count with peripheral smear and reticulocyte count, liver enzymes (ALT, AST), total bilirubin, serum creatinine, and estimated glomerular filtration rate (eGFR);
- Physical examinations;
- Vital signs monitoring;

Assessment of performance status, using the Karnofsky Performance Status (KPS) scale. Safety assessments are conducted prior to each PRRT cycle, 2 weeks after each cycle, and at least every 12 weeks during the follow-up period.

### 2.5 Preparation of [^177^Lu]Lu-DOTA-TATE and [^90^Y]Y-DOTA-TATE

[^177^Lu]Lu-DOTA-TATE and [^90^Y]Y-DOTA-TATE were prepared by radiolabeling DOTA-TATE (NCBJ POLATOM, Poland) with carrier-free ^177^LuCl_3_ (LutaPol, NCBJ POLATOM, Poland) or ^90^YCl_3_ (ItraPol, NCBJ POLATOM, Poland), respectively (17). Radiolabeling was carried out in sodium ascorbate buffer (pH 4.5) at 90°C for 30 minutes, followed by sterilization through a 0.22 μm filter. The final preparations were diluted with buffer to a concentration of 1.0 GBq/mL ±10% and dispensed into sterile vials under aseptic conditions.

### 2.6 Sample size determination and statistical analyses

Sample size estimation was based on the primary efficacy endpoint, progression-free survival (PFS), and comparison of survival curves using the log-rank test. An exponential distribution of PFS was assumed, with a baseline hazard rate in the standard arm of λ = 0.0235 per month, corresponding to a 20-month PFS rate of approximately 62–65%, consistent with the NETTER-1 trial (9). In Polish retrospective data (13), median PFS for tandem-PRRT ranged from 24.3 to 59.3 months depending on tumor grading. For the purpose of the DUONEN study, a 100% improvement in PFS was anticipated in the personalized PRRT arms, corresponding to a hazard ratio (HR) of 0.50.

Statistical analyses were performed using IBM SPSS Statistics software (version 23; IBM Corp., Armonk, NY, USA). The Shapiro–Wilk test was applied to assess the normality of data distribution. Normally distributed continuous variables are presented as means with standard deviations, whereas non-normally distributed variables are presented as medians with interquartile ranges (IQRs). Between-group comparisons were performed using the Kruskal–Wallis test for non-normally distributed data and analysis of variance (ANOVA) for normally distributed data, with *post-hoc* Tukey tests (equal variance) or Games–Howell tests (unequal variance) applied as appropriate. Correlations between variables were assessed using Pearson’s or Spearman’s correlation coefficients. Statistical significance was defined as a two-tailed p-value <0.05. P-values were not adjusted for multiple comparisons, as all analyses were exploratory.

## 3 Results

In the presented analysis, the labels T1, T2, T3, and T4 denote the first, second, third, and fourth cycles of PRRT, respectively. The labels V1, V2, V3, and V4 refer to the subsequent follow-up visits after the first (V1), second (V2), third (V3), and fourth (V4) cycle of PRRT.

### 3.1 Modifications of administered activities and organ doses

In the experimental arms B, C, and D, individualized dosimetry-guided modifications of administered activities were applied according to study protocol. By design, ^90^Y was modulated in arm B, ^177^Lu in arms C and D, while the other isotope (in arm B and C) was kept constant.

### 3.2 Patterns and magnitude of modifications

As summarized in **Table 2**, in arm B ^90^Y activity was reduced in 9 patients, increased in 2, and unchanged in 6, while ^177^Lu remained fixed. In arm C, ^177^Lu activity was reduced in 9 patients, increased in 1, and unchanged in 2, while ^90^Y remained fixed. In arm D, ^177^Lu was increased in 8 patients, decreased in 1, and unchanged in 3. Thus, activity reductions predominated in arms B and C, while increases were more common in arm D.

**Table 2:** Patterns of isotope modifications in arms B–D.

Arm	Isotope	N increased	N decreased	N unchanged	Mean ↑ (MBq, %)	Mean ↓ (MBq, %)
B	[^90^Y]	2	9	6	+2338 (+126%)	−1160 (−62.7%)
C	[^177^Lu]	1	9	2	+1055 (+28.5%)	−2532 (−68.4%)
D	[^177^Lu]	8	1	3	+1814 (+24.5%)	−2917 (−39.4%)

The magnitude of changes varied: average reductions amounted to −1160 MBq (−62.7%) for ^90^Y in arm B and −2532 MBq (−68.4%) for ^177^Lu in arm C, while increases averaged +2338 MBq (+126%) and +1055 MBq (+28.5%), respectively. In arm D, ^177^Lu activity was increased on average by +1814 MBq (+24.5%) and decreased by −2917 MBq (−39.4%).

**Figure 1:**
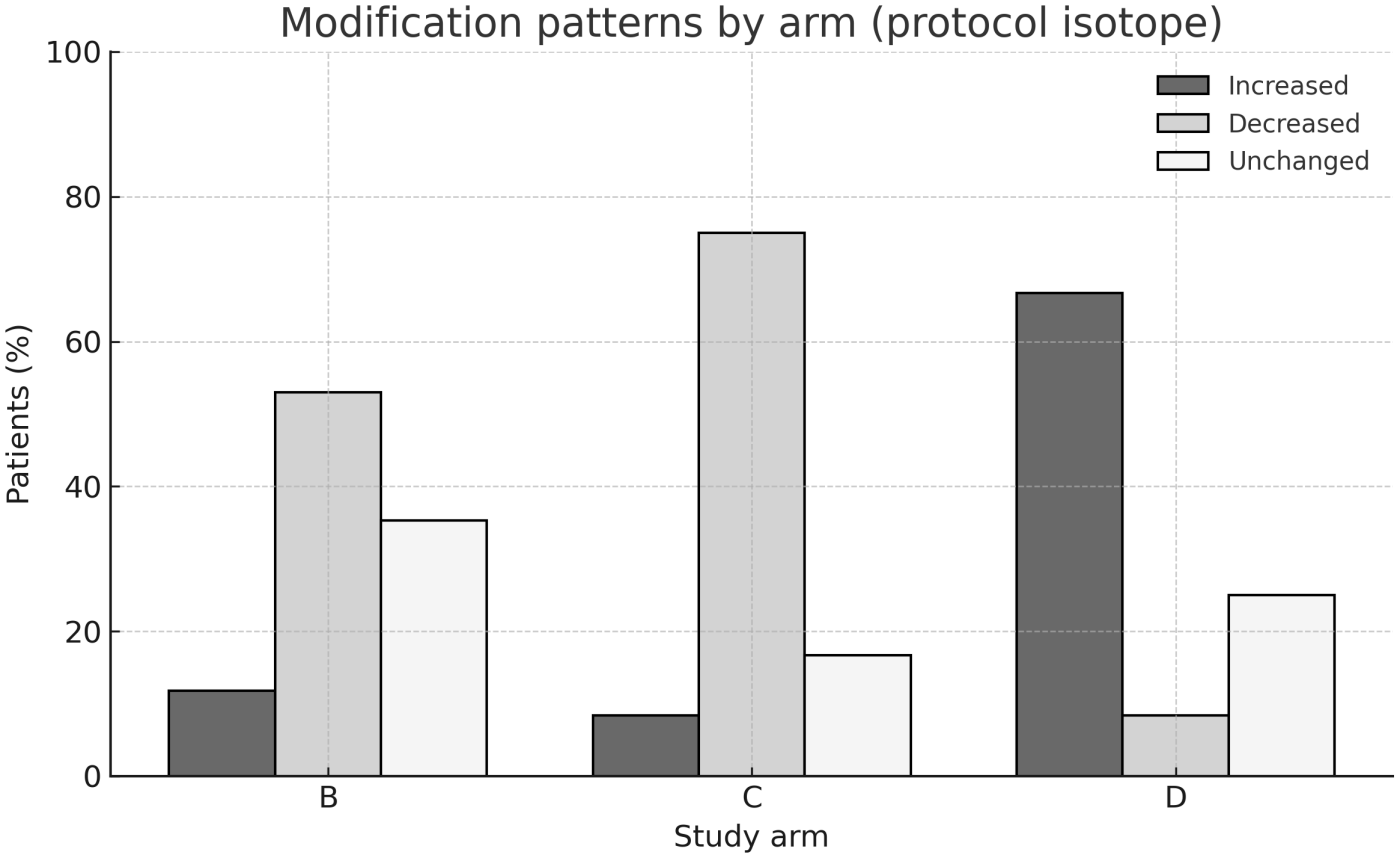
Modification patterns by study arm. Bar chart showing the proportion of patients with increased, decreased, or unchanged isotope activities in arms **(B-D)**. By protocol, [^90^Y] was modulated in arm **(B)** and [^177^Lu] in arms **(C, D)**, while the complementary isotope was kept constant. The majority of patients in arms **(B, C)** experienced activity reductions, whereas dose increases predominated in arm **(D)**.

These patterns are illustrated in **Figure 1**, which depicts the proportion of patients with increased, decreased, or unchanged activities per arm and isotope.

### 3.3 Impact on organ doses

Median cumulative kidney and marrow doses are presented in **Table 3**. Patients in the control arm A (fixed-activity ^177^Lu, 4×7.4 GBq) received the lowest doses to both kidneys (15.6 Gy, IQR 11.1–20.4) and marrow (0.39 Gy, IQR 0.25–0.57).

**Table 3:** Median cumulative organ doses per arm.

Arm	N	Kidney dose total, Gy (median, IQR)	kidney dose ^177^Lu, Gy	kidney dose ^90^Y, Gy	Marrow dose total, Gy (median, IQR)	marrow dose ^177^Lu, Gy	marrow dose ^90^Y, Gy
A	16	15.6 (11.1–20.4)	15.6	—	0.39 (0.25–0.57)	0.39	—
B	16	21.6 (17.1–26.2)	6.4	14.7	0.56 (0.42–0.71)	0.12	0.44
C	12	29.1 (21.6–36.2)	8.3	20.8	0.79 (0.59–1.13)	0.22	0.57
D	12	21.2 (17.4–23.9)	21.2	—	0.45 (0.37–0.52)	0.45	—

**Figure 2:**
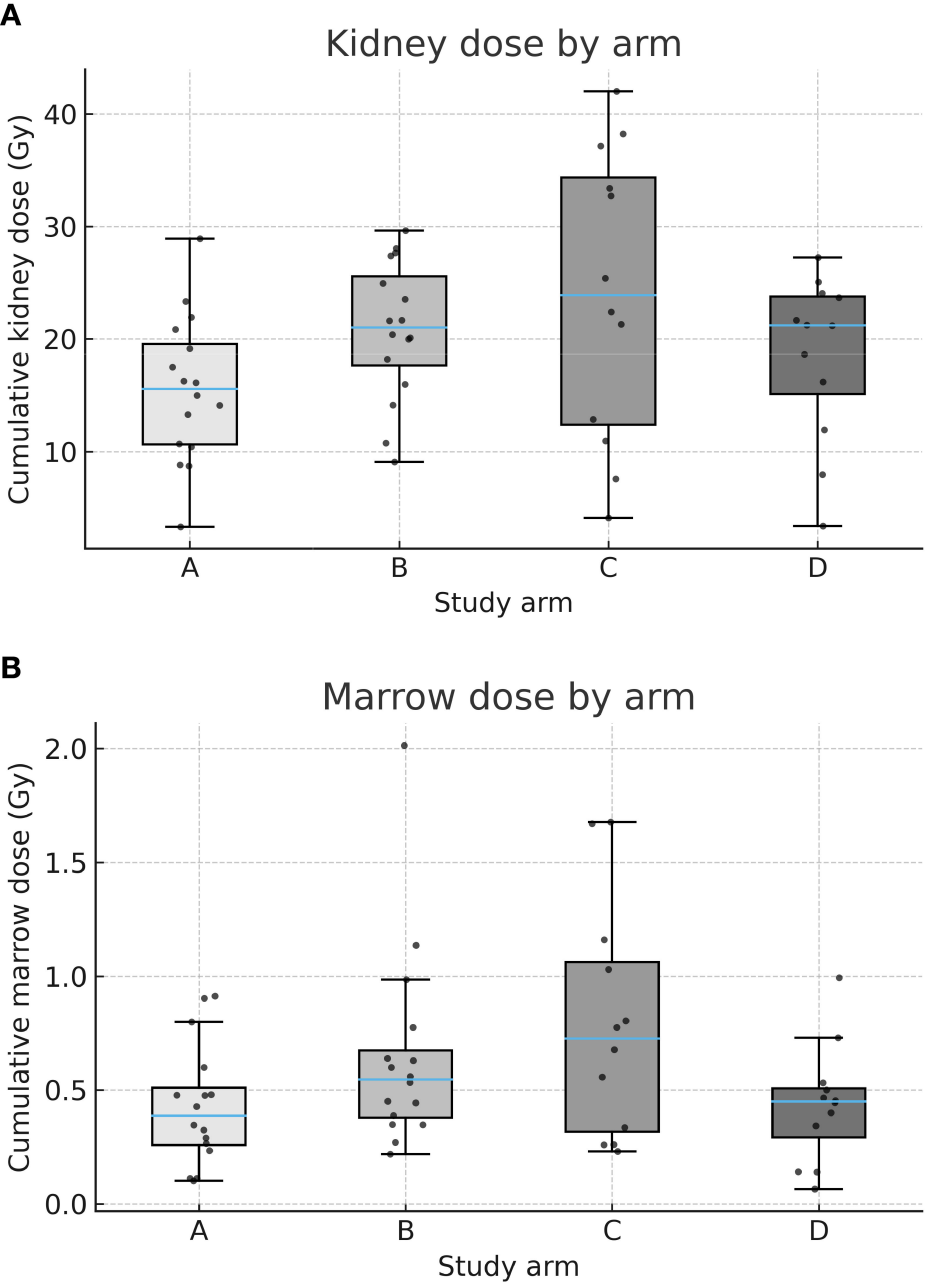
**(A)** Cumulative kidney dose by arm. Box-and-whisker plots (with jittered individual values) showing cumulative absorbed kidney doses (Gy) across arms **A–D**. Median kidney dose was lowest in arm A (fixed-activity [^177^Lu]) and highest in arm C (dosimetry-guided [^177^Lu] + fixed-activity [^90^Y]). **(B)** Cumulative marrow dose by arm. Box-and-whisker plots (with jittered individual values) showing cumulative absorbed marrow doses (Gy) across arms **A–D**. Median marrow dose was lowest in arm **A** and highest in arm **C**.

In comparison, kidney doses were higher in arms B (21.6 Gy, IQR 17.1–26.2) and C (29.1 Gy, IQR 21.6–36.2), with the highest marrow dose also observed in arm C (0.79 Gy, IQR 0.59–1.13). In arm D, median doses were 21.2 Gy (kidneys) and 0.45 Gy (marrow), comparable to those in arm B. Boxplots in **Figures 2A, B** illustrate the distribution of cumulative kidney and marrow doses per arm.

**Figure 3:**
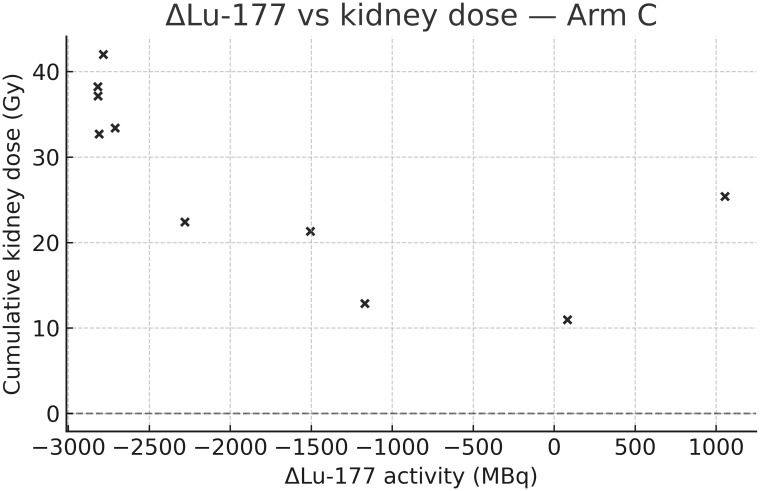
Correlation between Δ[^177^Lu] activity and kidney dose in arm C. Scatter plot showing the relationship between mean change in administered [^177^Lu] activity (post-baseline vs. baseline) and cumulative kidney dose (Gy) in arm C. A significant negative correlation was observed, indicating the predominant role of [^90^Y] in determining kidney exposure.

Importantly, in arms B and C, isotope-specific contributions could be distinguished. As shown in **Table 3**, ^90^Y accounted for the majority of renal dose in both arms, while marrow exposure remained modest. In arm C, a significant negative correlation was observed between changes in ^177^Lu activity and kidney dose (Spearman rho = −0.74, p=0.037), underscoring the dominant role of ^90^Y in determining renal exposure in tandem-RLT. This relationship is visualized in **Figure 3**.

### 3.4 Comparison with the standard arm

Statistical analyses confirmed higher kidney doses across arms (Kruskal-Wallis p=0.043), driven primarily by arm C (p=0.028 vs. arm A). Marrow doses were also higher in arm C compared to arm A (p=0.033), while arms B and D showed no significant differences.

Taken together, these results indicate that dosimetry-guided modifications were successfully implemented in the targeted isotopes, with reductions dominating in arms B and C and increases in arm D. Among the modified regimens, arm C yielded the highest renal and marrow doses relative to the standard fixed-activity protocol.

### 3.5 Hematologic toxicity over time

**Figure 4:**
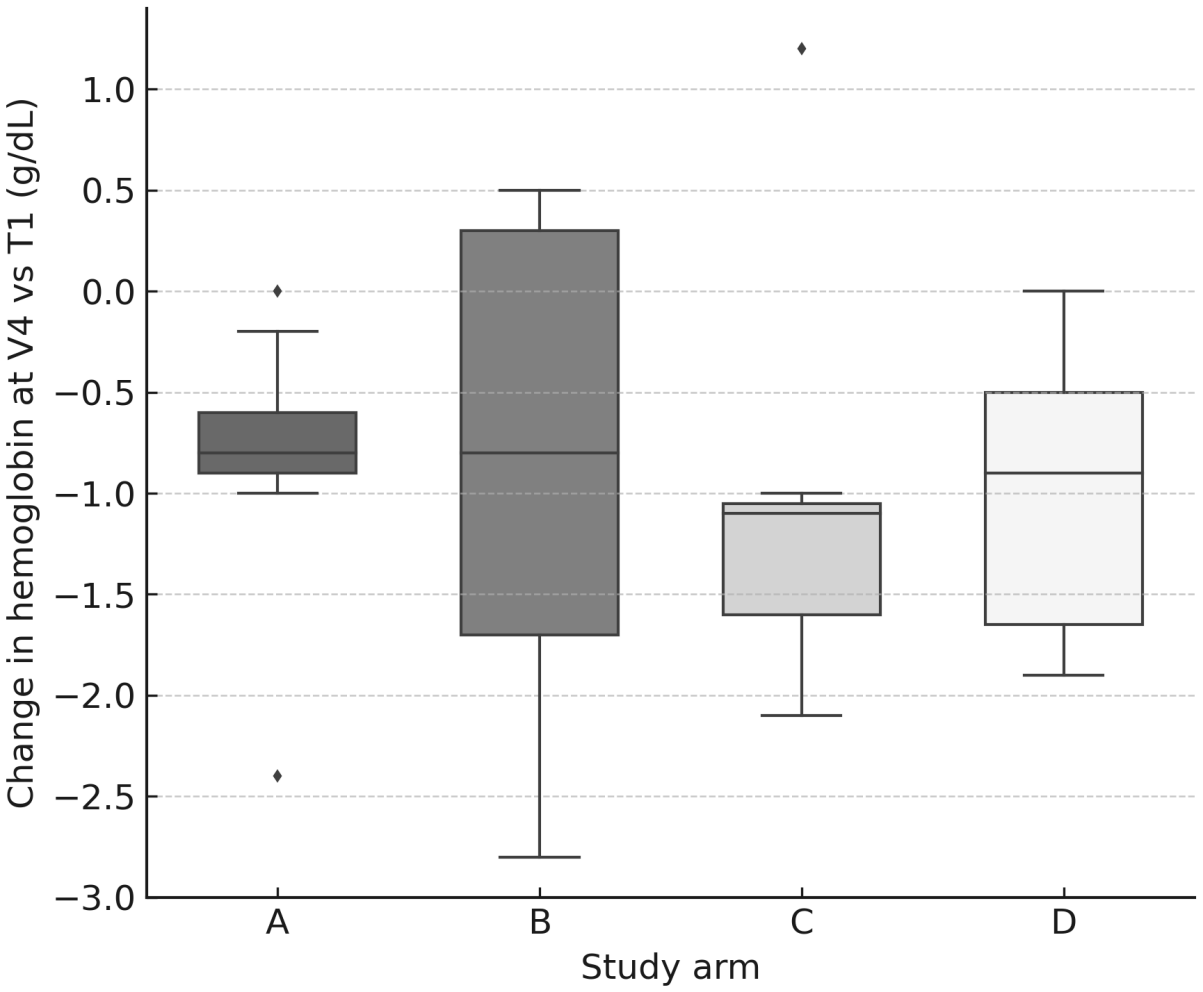
Hemoglobin change at V4 by arm. Box-and-whisker plots (with jittered individual values) showing change in hemoglobin concentration (g/dL) at V4 relative to baseline (T1). A modest but consistent decline was observed across all arms.

**Figure 5:**
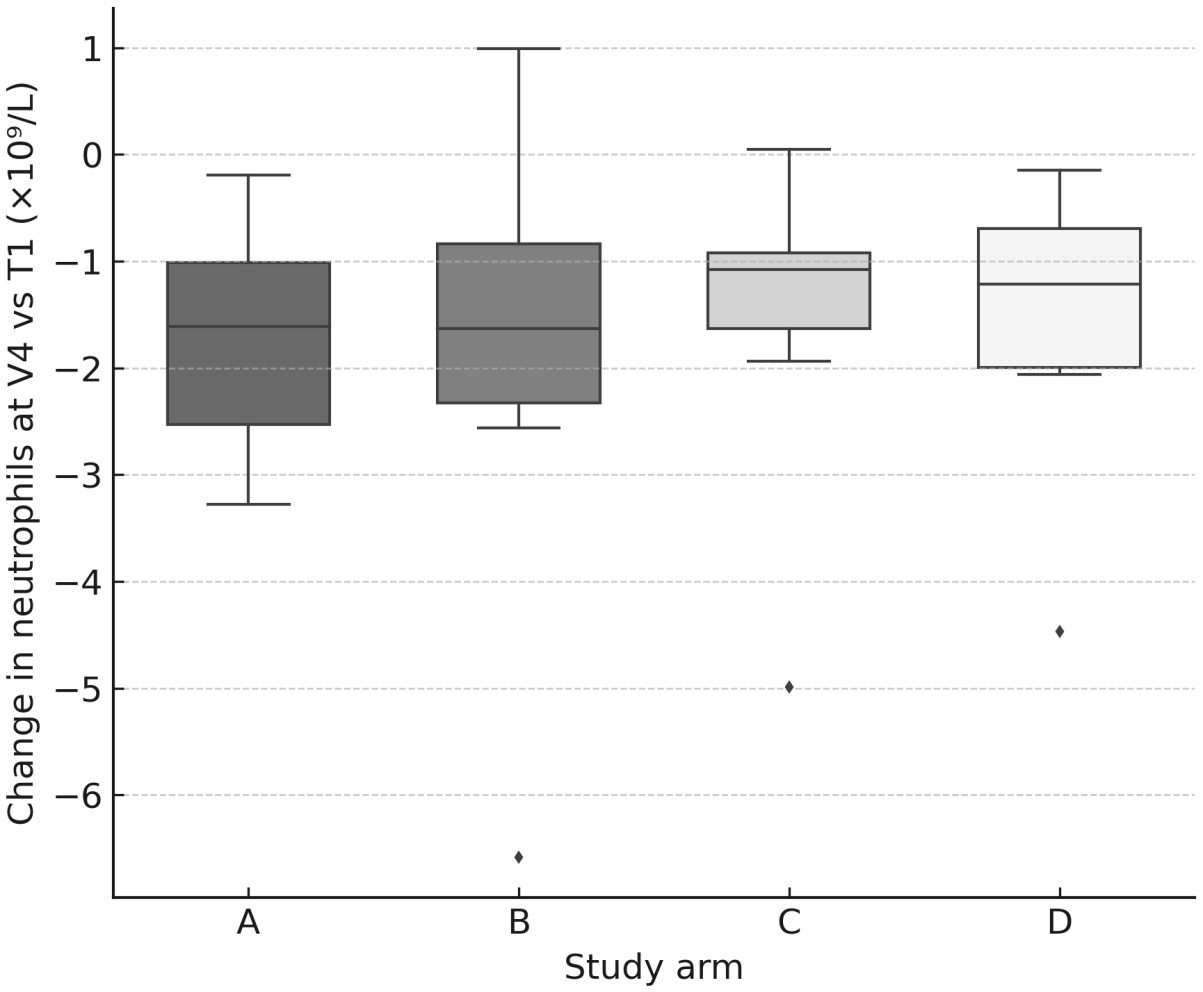
Neutrophil change at V4 by arm. Box-and-whisker plots (with jittered individual values) showing change in neutrophil count (×10^9^/L) at V4 relative to baseline (T1). Declines were observed in all arms, consistent with transient hematologic toxicity.

Across the cohort, blood counts declined progressively after consecutive PRRT cycles, with the largest median drops (vs T1 baseline) observed at V4 (**Figures 4**, **5**): hemoglobin −0.90 g/dL (IQR −1.60 to −0.50), platelets −57×10^9^/L (−88 to −22), WBC −2.52×10^9^/L (−3.38 to −1.72), neutrophils −1.37×10^9^/L (−2.06 to −0.87), and lymphocytes −0.87×10^9^/L (−1.31 to −0.53) (**Table 4**). Paired comparisons showed stepwise, statistically significant declines between early visits: V1→V2 for Hb (p=0.0017), PLT (p=0.0001), WBC (p<0.0001), NEU (p=0.0023), LYM (p<0.0001), and also V2→V3 for Hb (p=0.013), PLT (p=0.0026), WBC (p=0.023), NEU (p=0.17 ns), LYM (p=0.0049). Additional decline in PLT was seen for V3→V4 (p=0.016).

**Table 4:** Safety summary (Δ vs T1).

Analyte	Visit of largest median Δ	Median Δ (IQR)	Between-arm p<0.05 (visits)
Hemoglobin	V4	−0.90 g/dL (−1.60 to −0.50)	V1 (p=0.027)
Platelets	V4	−57×10^9^/L (−88 to −22)	V3 (p=0.047)
WBC	V4	−2.52×10^9^/L (−3.38 to −1.72)	—
Neutrophils	V4	−1.37×10^9^/L (−2.06 to −0.87)	—
Lymphocytes	V4	−0.87×10^9^/L (−1.31 to −0.53)	—
Creatinine	V2 (worst ↑ would be +; here ↓)	−0.08 mg/dL (−0.15 to 0.00)	V2 (p=0.036), V3 (p=0.013)
eGFR	V4	+1.70 mL/min/1.73 m² (−1.00 to 12.95)	V3 (p=0.0078), V4 (p=0.0035)
AlAT	V3	−5.5 U/L (−10.3 to 1.25)	—
AspAT	V1	−2.0 U/L (−7.25 to 0.25)	—
Bilirubin	V1	−0.047 mg/dL (−0.123 to 0.10)	—

median Δ with IQR; visit with largest median drop; significant between-arm signals where present; T1–T4 = treatment cycles (first to fourth RLT cycle); V1–V4 = follow-up visits after each corresponding cycle; WBC - white blood count; eGFR - estimated glomerular filtration rate; ALT - alanine aminotransferase; AST - aspartate aminotransferase.

Between-arm differences in Δ vs T1 were limited: Kruskal–Wallis reached p<0.05 for Hb at V1 (p=0.027) and PLT at V3 (p=0.047), without a consistent pattern across timepoints. Notably, dose modifications (↑/↓ vs unchanged) in arms B–D did not significantly shift hematologic nadirs in within-arm tests (all p≥0.05).

**Figure 6:**
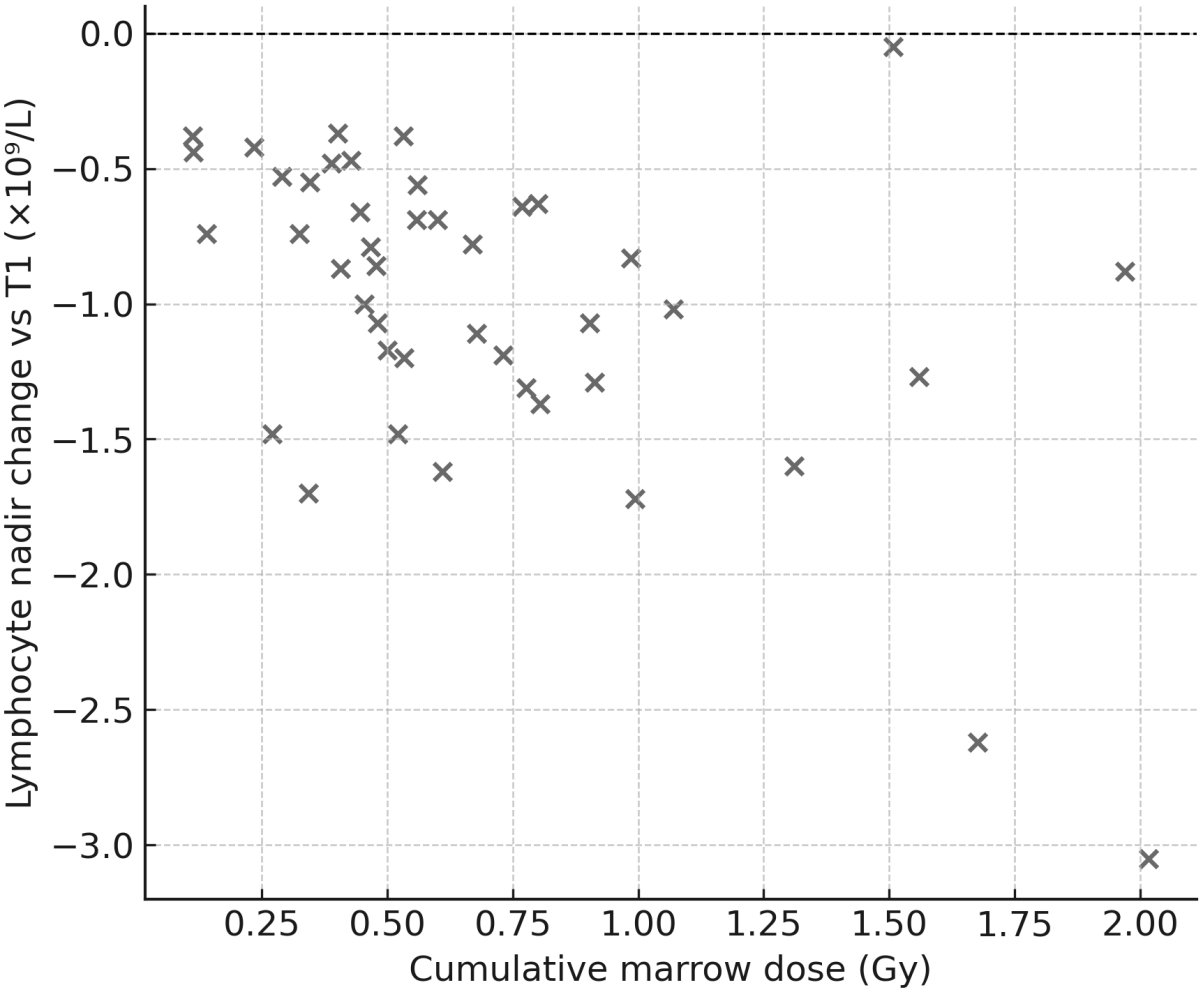
Lymphocyte nadir vs. cumulative marrow dose. Scatter plot showing the correlation between nadir lymphocyte count (Δ vs. T1) and cumulative marrow dose (Gy). A moderate negative correlation was identified (Spearman ρ≈−0.49), indicating that greater marrow exposure was associated with deeper lymphocyte declines.

Dose–effect relationship (marrow): hematologic nadirs correlated with cumulative marrow dose: Hb (ρ=−0.40, p=0.0047), WBC (ρ=−0.37, p=0.010), LYM (ρ=−0.49, p=0.0006). Thus, deeper drops occurred in patients with higher marrow exposure (**Figure 6**).

### 3.6 Renal function over time

**Figure 7:**
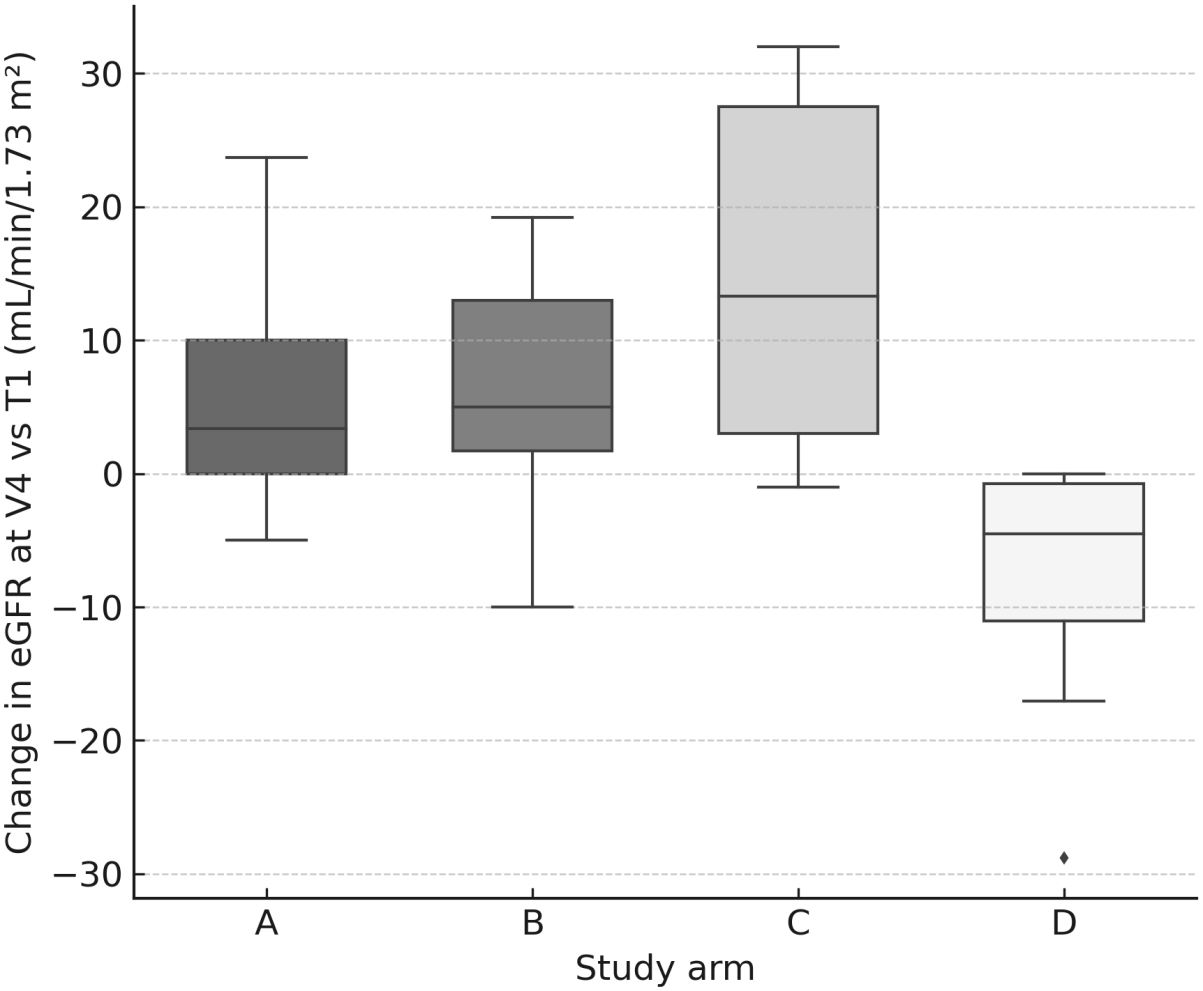
eGFR change at V4 by arm. Box-and-whisker plots (with jittered individual values) showing change in estimated glomerular filtration rate (mL/min/1.73 m²) at V4 relative to baseline (T1). No clinically significant differences were observed between arms.

Renal biochemistry remained stable overall. The worst median Δ creatinine occurred at V2 and was negative (−0.08 mg/dL; IQR −0.15–0.00), and eGFR medians tended to be stable to slightly improved by V4 (+1.70 mL/min/1.73 m²; IQR −1.00–12.95). Paired tests showed V3→V4 changes for creatinine (p=0.011) and eGFR (p=0.001), consistent with mild late shifts but without clinical deterioration. Between-arm tests detected differences at selected visits (creatinine V2 p=0.036, V3 p=0.013; eGFR V3 p=0.0078, V4 p=0.0035), yet changes in creatinine/eGFR did not correlate with cumulative kidney dose (no significant Spearman correlations), and activity-modification categories (↑/↓/=) in B/C/D did not significantly affect renal **“**worst Δ**”** in within-arm analyses. Importantly, arm D (escalated ^177^Lu) showed no signal of worsened creatinine/eGFR vs other arms at V4 (**Figure 7**).

### 3.7 Hepatic parameters

ALT, AST and bilirubin exhibited small negative median shifts (e.g., ALT V3 −5.5 U/L) without consistent between-arm differences and without significant paired changes indicative of hepatotoxicity.

### 3.8 Interpretation focused on trial questions

- Arm D (higher ^177^Lu activities): higher ^177^Lu vs baseline did not worsen blood counts or renal function, and marrow/renal doses remained within safety bounds.
- Arms B/C (tandem settings, especially C): although kidney and marrow doses were higher (driven chiefly by ^90^Y), this did not translate into disproportionate creatinine/eGFR worsening; hematologic declines correlated with marrow dose rather than with the categorical decision to escalate/de-escalate activity.

### 3.9 Adverse events

In total, 38 adverse events (AEs) were reported during the active treatment phase (**Table 5**). The majority were of mild or moderate intensity (CTCAE grade 1–2). Overall, 5 events (13.2%) were grade ≥3, with no persistent high-grade hematological toxicities observed. Hematological abnormalities were the most frequently reported events, including anemia (n=14, 36.9%), thrombocytopenia (n=11, 28.9%), and leukopenia/neutropenia (n=4, 10.6%). No cases of myelodysplastic syndrome were observed in any of the patients participating in the study. In addition, renal impairment (n=4, 10.6%), primarily increases in serum creatinine and decreases in eGFR, was documented, all grade 1–2. No cases of permanent renal failure were observed among the study participants, and none of the patients required renal replacement therapy. Single cases of alopecia, fatigue, and gastrointestinal complaints such as nausea, diarrhea or abdominal discomfort were observed, but each occurred only sporadically. All 5 event of grade ≥3 required discontinuations of the PRRT. Overall, the safety profile during the active treatment period was consistent with expectations, with adverse events being largely predictable, manageable, and reversible with supportive care.

**Table 5:** Adverse events reported during RLT.

Category	All reported events (N, %)	Events grade ≥3 (N, %)
Anemia	14 (36.9%)	0 (0%)
Thrombocytopenia	11 (28.9%)	5 (13.2%)
Leukopenia/neutropenia	4 (10.6%)	0 (0%)
Renal impairment	4 (10.6%)	0 (0%)
Alopecia	1 (2.6%)	0 (0%)
Fatigue	1 (2.6%)	0 (0%)
Diarrhea	1 (2.6%)	0 (0%)
Nausea	1 (2.6%)	0 (0%)
Abdominal discomfort	1 (2.6%)	0 (0%)
Total	38 (100%)	5 (13.2%)

## 4 Discussion

The DUONEN is the first in Poland and one of the few worldwide, an ongoing, prospective, randomized clinical trial comparing standard peptide receptor radionuclide therapy (PRRT) based on fixed-activity dose of [^177^Lu]Lu-DOTA-TATE with dosimetry-guided PRRT strategy in which activities were adapted after each treatment cycle. This pre-specified interim analysis was conducted after more than 60% of the planned cohort (56 of 92 patients) had completed the active phase of PRRT. Because follow-up after PRRT is not yet complete, this interim analysis does not focus on objective efficacy endpoints such as progression-free survival (PFS) or overall survival (OS). Instead, the purpose of this report is to confirm the safety of the ongoing randomized clinical trial and to validate the assumptions underlying the DUONEN trial design. The results obtained so far provide several important insights relevant to the further development of PRRT in neuroendocrine tumors.

### 4.1 Dosimetry and activity modifications

As demonstrated, the use of individualized dosimetry in arms B, C, and D led to both upward (predominantly in arm D) and downward (predominantly in arms B and C) activity adjustments. Notably, in arm D (where after the first cycle of 7.4 GBq [^177^Lu]Lu-DOTA-TATE, activities for subsequent cycles were modified individually based on dosimetry), higher-than-standard PRRT activities could be safely administered in 8 of 12 patients (66.7%). Similar findings were reported by Sandström et al. (11), who showed that over 50% of patients in their 200-patient cohort treated with standard fixed 7.4 GBq [^177^Lu]Lu-DOTA-TATE could have continued treatment safely beyond four cycles, as critical organ dose limits (kidneys and bone marrow) were not reached. In their study, the adopted strategy was to escalate the number of treatment cycles if safety limits were not exceeded, yielding a median of 4.53 cycles (range 2–10; IQR 3.87–5.52), corresponding to a median cumulative administered activity of 33.52 GBq. In our analysis, the median cumulative activity in arm D was 31.28 GBq (range 14.26–48.67; IQR 24.72–32.59). In our analysis, where activity was modified per cycle, and in the Sandström et al. study, where the number of fixed-activity cycles was varied, no increase in hematologic toxicity or renal impairment was observed in patients receiving cumulative activities above the standard 29.6 GBq. This provides an important argument for a more flexible approach to RLT planning. Of course, the critical question remains whether activity escalation (or increasing the number of cycles, as in Sandström et al.) will translate into improved efficacy. Such analyses are not yet available in DUONEN and will be the subject of future reports. In a dosimetry report from the NETTER-1 trial published in 2025 (18), the median cumulative absorbed kidney dose in a cohort of 20 patients was 19.3 Gy, remaining below the predefined threshold of 23 Gy; only three patients in this group treated with standard [^177^Lu]Lu-DOTA-TATE PRRT exceeded this limit. In our cohort of patients treated with standard PRRT (Group A), the median absorbed kidney dose was 15.6 Gy, and none of the 14 analyzed patients exceeded the threshold of 23 Gy.

In arms B and C, which employed so-called tandem-PRRT combining [^177^Lu]Lu-DOTA-TATE and [^90^Y]Y-DOTA-TATE in variable proportions, higher absorbed doses in critical organs (kidneys and bone marrow) were observed, mainly driven by the biodistribution of [^90^Y]Y-DOTA-TATE. These findings are consistent with earlier Polish and European retrospective reports (13, 14, 16). Polish experiences (13, 14) emphasized that although tandem-PRRT may increase exposure to critical organs, it may simultaneously enhance efficacy in patients with bulky tumors or heterogeneous metastatic disease. The main advantage of DUONEN results is that they originate from a prospective trial in which isotope activities in tandem arms were individualized according to dosimetry. Unlike prior retrospective studies, where the ratio of [^177^Lu]Lu-DOTA-TATE to [^90^Y]Y-DOTA-TATE was fixed at 1:1, in DUONEN the ratio in the first cycles of arms B and C was set at 2:1 (3.7 GBq [^177^Lu]Lu-DOTA-TATE and 1.85 GBq [^90^Y]Y-DOTA-TATE), better reflecting the differences in radiation energy of the two isotopes. Importantly, in both tandem arms, activities of the modifiable isotope (^90^Y in arm B, ^177^Lu in arm C) were reduced in most patients (9 of 16 (56.3%) and 9 of 12 (75.0%), respectively). This may suggest that despite the adjusted 2:1 ratio, the activities in many patients were still too high for a fixed four-cycle regimen. To date, no randomized phase III trials have reported on the safety and efficacy of dosimetry-based tandem-PRRT using somatostatin analogs labeled with ^90^Y. U.S. phase II data (19) on [^90^Y]Y-DOTA-TOC are available, where the first course was administered at 4.4 GBq, and subsequent activities were adjusted based on dosimetry to 1.7–5.6 GBq. In that study, 3 of 25 patients discontinued after the first course, while among the remaining 22 patients, subsequent activities were reduced or maintained in 45%. Again, it should be emphasized that the impact of different PRRT regimens on PFS and OS represents one of the main objectives of the DUONEN trial, and these analyses will be available and reported once the entire study cohort has completed treatment and follow-up. It is worth emphasizing that the dosimetric findings presented in this interim analysis are consistent with the preliminary report conducted in a smaller subset of DUONEN patients (64 administrations in 36 patients), which aimed to validate the dosimetric methodology applied in this trial (20). The stability of the previously reported results, despite the enlargement of the analyzed cohort and the extended study duration, confirms the robustness of the methodological approach adopted in DUONEN and supports the continued implementation of the study protocol.

### 4.2 Hematologic and renal safety

Safety analyses indicate predictable hematologic declines that increased with subsequent PRRT cycles across all study arms. The most pronounced decreases were observed in lymphocytes and platelets, consistent with earlier publications. In the Polish study by Saracyn et al. (21), conducted in a cohort of 42 patients treated with either standard PRRT or tandem-PRRT, a significant decrease in all hematological parameters was observed when comparing results between the first and the fourth treatment course. In NETTER-1, the first randomized trial assessing efficacy of fixed-activity [^177^Lu]Lu-DOTA-TATE PRRT in GEP-NET patients, worsening of hematologic parameters was primarily seen in white blood cell and platelet counts (9). In that trial (design analogous to our arm A), the majority of patients (77%) in the [^177^Lu]Lu-DOTA-TATE group received all four planned infusions, and eight patients required activity reduction. Similarly, in our study, PRRT in arm A was discontinued before the fourth cycle in 3 of 16 patients (81.3% completed treatment as planned). A comparable outcome was seen in arm D, where variable activities of [^177^Lu]Lu-DOTA-TATE were used - 10 of 12 patients (83.3%) completed all four cycles. Importantly, activity modifications in arms B, C, and D did not result in statistically significant changes in nadir hematologic values in within-arm comparisons.

Most severe, long-term hematologic complications (persistent hematologic disorders, PHD) described in the literature occur months to years after PRRT. Bergsma et al. (22) reported a prevalence of 4% PHD after [^177^Lu]Lu-DOTA-TATE, with a median time to onset of 41 months. The current interim analysis of DUONEN includes only early post-treatment hematologic results. The full protocol includes a 5-year follow-up, and late hematologic events will be reported subsequently, however it should be emphasized that during the period covered by the analysis (as well as throughout the subsequent course of the DUONEN study, which is not included in the present analysis), no cases of myelodysplastic syndrome were observed in any of the patients participating in the study.

An interesting finding was the lack of statistically significant differences between arms in the depth of hematologic declines, despite higher marrow doses in arms B and C compared to arm A. As noted, in arms B and C, activities in subsequent cycles were frequently reduced, primarily due to approaching the renal dose limit rather than the marrow threshold. Indeed, in most patients in this interim analysis, renal dose was the limiting factor for reducing PRRT activity. Nevertheless, renal function remained stable across all study arms, and observed inter-arm differences were not clinically significant. Premature treatment discontinuations were usually related to hematologic rather than renal toxicity. These findings suggest that the traditionally applied renal dose limit of 23 Gy remains safe even in the context of dosimetry-driven therapy. It should be emphasized, however, that both the 23 Gy kidney and 2 Gy marrow limits were historically derived from external beam radiotherapy (23) and ^131^I therapy (24). Growing evidence suggests these thresholds may be overly conservative in the PRRT setting. Large cohorts treated with [^177^Lu]Lu-DOTA-TATE have shown clinically relevant nephrotoxicity to be rare even at doses above 23 Gy (11, 25), and true safety limits may be higher, particularly when expressed as biologically effective dose (BED) (26). Similarly, the 2 Gy marrow threshold is based on simplified blood-based models (23), while more recent image-based studies suggest a more complex dose–effect relationship with hematologic toxicity (27). In DUONEN, activities in arms B, C, and D were adjusted in cycles 2–4 to approach but not exceed these thresholds. In arm C, however, the fixed use of 1.85 GBq [^90^Y]Y-DOTA-TATE in each cycle, with only [^177^Lu]Lu-DOTA-TATE adjusted, resulted in renal dose thresholds being exceeded in some patients. Nonetheless, no early renal toxicity was observed, indirectly supporting the concept that the 23 Gy kidney threshold derived from external beam radiotherapy may be conservative for PRRT.

It should also be noted that no significant hepatological adverse events were observed in any of the four study arms, either in cycles using pure ^177^Lu or in tandem-PRRT. These observations are consistent with previously published data (28). Although those reports are derived from retrospective studies, they, similarly to the DUONEN trial, included patients treated with both ^177^Lu-based PRRT and tandem-PRRT. Furthermore, the available literature indicates that the liver is not considered a critical organ limiting the use of PRRT (18). Nevertheless, given the very high prevalence of hepatic metastases in patients with GEP-NETs, the potential for increased radiation exposure to liver tissue must be acknowledged. Therefore, in the DUONEN study, biochemical liver function parameters are systematically assessed both during qualification for standard and dosimetry-guided PRRT, as well as throughout follow-up.

### 4.3 Clinical significance and practical implications

Our early observations carry several clinical implications. First, standard PRRT (arm A) remains safe and predictable, but does not allow treatment individualization, which does not align with the current paradigm of personalized medicine. A significant proportion of patients may not reach critical organ thresholds, leaving room for potential escalation of activity or number of cycles. Second, arm D demonstrates that escalation of [^177^Lu]Lu-DOTA-TATE activity in selected patients is feasible, potentially beneficial, and safe, paving the way for future studies on maximizing efficacy while maintaining safety. Third, arms B and C illustrate that tandem-PRRT regimens (with added [^90^Y]Y-DOTA-TATE) increase absorbed doses in critical organs, necessitating caution and long-term follow-up, but may be advantageous in patients with bulky or poorly penetrated lesions. Literature data support these assumptions, with Baum et al. (16) reporting the longest OS in retrospective analyses of 1048 patients treated with various PRRT regimens for those receiving tandem-PRRT (64 months vs 44 months for [^177^Lu]Lu-DOTA-TATE alone). Aalbersberg et al. similarly reported longer OS and PFS with tandem-RLT, though noting potential selection bias, as patients with higher tumor burden were more likely to receive dual-isotope regimens (29). Importantly, our randomized design avoids this limitation, as patients with both large and small metastases were included in arms B and C. Final OS and PFS analyses will be reported upon study completion. Prospective, randomized confirmation is particularly relevant in light of recent retrospective Polish data (Durma et al.), which in a cohort of 51 other than midgut and pancreatic NET patients (30) and in a cohort of 167 midgut and pancreatic NET patients (31) found no significant OS or PFS differences between treatment subgroups based on the PRRT regimen used. However, both studies were retrospective and non-randomized in treatment allocation, and one of them included patients with NETs other than midgut and pancreatic primaries, whereas the majority of patients in the DUONEN trial had midgut or pancreatic NETs. It should also be taken into consideration that non-pancreatic NETs, such as those originating from the lung or rectum, may display a more aggressive clinical course. Moreover, one of that study cohort included patients with NET G3, who were not eligible for inclusion in the DUONEN trial.

Although the DUONEN study comprises four treatment arms, all regimens are based exclusively on β^-^-emitting radionuclides. In recent years, the concept of PRRT using α-particle emitters (α-PRRT, also referred to as targeted alpha therapy, TAT) such as actinium-225 (^225^Ac), bismuth-213 (^213^Bi), or lead-212 (^212^Pb) (32) has gained increasing attention. However, due to the limited global production capacity of alpha emitters, clinical trials using these isotopes are still restricted to small patient cohorts, and widespread adoption of α-PRRT is unlikely in the near future. Alpha particles, characterized by high linear energy transfer (LET) and a short tissue penetration range (<100 μm), induce highly localized DNA damage and allow effective eradication of micrometastatic or poorly vascularized tumor clusters. Encouraging results from early clinical experiences have demonstrated the efficacy of α-PRRT, including prolonged PFS and objective responses in patients refractory to standard (β^-^-based) PRRT (33–35). These findings have provided the rationale for exploring combined α/β tandem-PRRT approaches. Most reports on such therapies originate from German centers (36, 37), where simultaneous administration of ^177^Lu and ^225^Ac was performed, frequently using somatostatin receptor antagonists, which exhibit markedly higher receptor affinity (32, 38) than the agonists used in conventional PRRT and in the DUONEN protocol. Optimization of dosing schemes, sequencing, and radiobiological modeling remains critical to balancing efficacy and safety, particularly regarding renal and hematologic toxicity. Nevertheless, individual activity tailoring in α-PRRT remains technically challenging - if not impossible - because of the phenomenon of radioactive decay chains producing daughter isotopes (39, 40). These isotopes, which are themselves α-emitters with distinct energies, half-lives, and ligand affinities, may lead to unpredictable pharmacokinetics, biological effects, and toxicity, rendering accurate dosimetry difficult or unfeasible (39). Ongoing studies are expected to better define the clinical role of α-based tandem PRRT and its integration into personalized theranostic strategies.

### 4.4 Limitations and strengths

The findings of this interim analysis should be considered in light of several important limitations. First, the presented results are based on an interim evaluation, and long-term efficacy endpoints such as progression-free survival (PFS) and overall survival (OS) are not yet available. Consequently, the findings should be primarily interpreted in terms of dosimetry and safety rather than treatment efficacy. Second, although the study was randomized and multicenter, the overall sample size remains limited and suboptimal, which reduces the power of between-arm comparisons. The sample size calculation assumed a two-sided significance level (α = 0.05), 90% statistical power, uniform patient accrual over five years, and a total study duration of ten years. For the primary comparison combining all personalized PRRT arms (B + C + D) versus the standard PRRT arm (A) with a 3:1 allocation ratio, approximately 117 PFS events were required to achieve 90% power. Under the assumed event rate of ~70%, this corresponds to an overall sample size of about 167 patients (approximately 42 per arm). Due to funding constraints, the study was planned to include 92 patients (23 per arm). This sample size ensures adequate precision for safety assessments (for instance, a 10% incidence of grade ≥3 renal toxicity can be estimated with a 95% CI half-width of approximately 6%) and provides exploratory power for efficacy analyses. For the pooled comparison (B + C + D vs. A), the expected statistical power is approximately 33% for HR = 0.667, 75% for HR = 0.50, and 89% for HR = 0.43. Pairwise comparisons between individual experimental arms and the control arm will be treated as exploratory and presented with 95% confidence intervals without adjustment for multiplicity. Third, all statistical analyses were exploratory; p-values were not adjusted for multiple testing, and results should therefore be interpreted with caution. Fourth, conventional organ dose limits of 23 Gy for kidneys and 2 Gy for bone marrow were applied as reference thresholds. These limits originate from external beam radiotherapy and ^131^I therapy and may be conservative in the PRRT context; nevertheless, they remain the most widely accepted benchmarks in clinical practice. Emerging evidence suggests that higher dose thresholds may be tolerable in PRRT, but a definitive consensus has not yet been established. Fifth, although safety monitoring included hematologic and renal parameters, rare but clinically significant adverse events - such as grade ≥3 toxicities or treatment discontinuations due to toxicity - warrant continued surveillance in the final analysis.

Despite these limitations, the study has several notable strengths. DUONEN represents the first randomized, multicenter trial directly comparing fixed-activity [^177^Lu]Lu-DOTA-TATE with multiple predefined dosimetry-guided strategies, including ^90^Y/^177^Lu tandem therapy, with per-cycle activity adjustment. The systematic dosimetry performed after each cycle allowed for quantification of isotope-specific organ contributions, providing novel prospective evidence that has not been available from retrospective series or single-arm trials. This is particularly important, as the existing literature explicitly indicates the lack of randomized controlled clinical trials on dosimetry-guided PRRT (41, 42). The DUONEN study fills this gap in an excellent way. Moreover, the multicenter design enhances the generalizability of the findings across different nuclear medicine departments. Finally, the comprehensive safety evaluation—including both dosimetric endpoints and laboratory toxicity - provides a robust foundation for refining PRRT protocols and for informing future guidelines on individualized, dosimetry-driven treatment. To emphasize the importance of implementing dosimetry in PRRT, it is worth noting that during the annual congress of the European Association of Nuclear Medicine (EANM) held in October 2025 in Barcelona, Spain the prestigious Marie Curie Award was granted to an international research team for conducting patient-specific dosimetry within the COMPETE trial (43, 44). Notably, the dosimetric analysis in COMPETE was performed in a cohort of only 20 patients - substantially smaller than the group presented in the current study - further underscoring the significance and scale of the dosimetric work achieved in our investigation.

## 5 Conclusions

In conclusion, DUONEN provides the first randomized, multicenter evidence directly comparing fixed-activity PRRT with several predefined dosimetry-guided strategies, including ^90^Y/^177^Lu tandem therapy with per-cycle adaptation. While these interim results are limited to safety and dosimetry, they demonstrate the feasibility and safety of individualized, dosimetry-driven protocols and highlight their potential to refine established kidney and marrow dose thresholds. Larger studies with longer follow-up will be required to confirm the impact of these approaches on long-term efficacy outcomes.

## Data Availability

The original contributions presented in the study are included in the article/**Supplementary Material**. Further inquiries can be directed to the corresponding author.
