# Supplementary material for: Dosimetry-guided peptide receptor radionuclide therapy in neuroendocrine tumors: interim safety analysis of the DUONEN trial
Source: Front Endocrinol (Lausanne). 2025 Dec 1;16:1716247. doi: 10.3389/fendo.2025.1716247 (PMC12702747; doi:10.3389/fendo.2025.1716247)
Supplement: Supplementary file 1 [file Table1.docx]

**CONSORT 2010 Checklist – DUONEN Interim Analysis**

| Section/Item | CONSORT 2010 Checklist  Item | Reported on page / section |
| --- | --- | --- |
| Title and Abstract | 1a. Identification as a randomized trial in the title | Title page – Dosimetry-Guided Radioligand Therapy in Neuroendocrine Tumors: Interim Safety Analysis of the DUONEN Trial |
| Title and Abstract | 1b. Structured summary of trial design, methods, results, and conclusions | Abstract – Structured with Background, Methods, Results, Conclusions |
| Introduction | 2a. Scientific background and explanation of rationale | Introduction, p. 3–4 |
| Introduction | 2b. Specific objectives or hypotheses | Introduction, last paragraph – Aim of DUONEN trial |
| Methods – Trial design | 3a. Description of trial design (parallel, factorial, etc.), allocation ratio | Methods, Trial design – multicenter, randomized, four arms, allocation ratio 1:1:1:1 |
| Methods – Trial design | 3b. Important changes to methods after trial commencement, with reasons | Not applicable – no changes reported |
| Methods – Participants | 4a. Eligibility criteria for participants | Methods, Eligible patients – inclusion and exclusion criteria |
| Methods – Participants | 4b. Settings and locations where data were collected | Methods – four clinical centers in Poland |
| Methods – Interventions | 5. Interventions for each group with sufficient detail to allow replication | Methods, Trial design – arms A–D described in detail |
| Methods – Outcomes | 6a. Pre-specified primary and secondary outcome measures, including how and when assessed | Methods, RLT safety evaluation – primary focus on safety (hematology, renal, hepatic) |
| Methods – Outcomes | 6b. Changes to trial outcomes after trial commenced, with reasons | Not applicable – interim safety analysis only |
| Methods – Sample size | 7a. How sample size was determined | Methods – planned N=92, rationale not fully described in interim paper (protocol reference) |
| Methods – Sample size | 7b. When applicable, explanation of interim analyses and stopping guidelines | Methods – pre-specified interim analysis after 56/92 patients completed active phase |
| Methods – Randomisation | 8a. Method used to generate random allocation sequence | Methods – Randomization mentioned, but specific method not detailed (refer to protocol) |
| Methods – Randomisation | 8b. Type of randomisation; details of any restriction (blocking, stratification) | Not detailed in manuscript – in protocol |
| Methods – Allocation concealment | 9. Mechanism used to implement the random allocation sequence | Not detailed in manuscript – in protocol |
| Methods – Implementation | 10. Who generated the sequence, who enrolled participants, and who assigned interventions | Not detailed in manuscript – in protocol |
| Methods – Blinding | 11a. If done, who was blinded and how | Open-label trial – not blinded |
| Methods – Blinding | 11b. If relevant, similarity of interventions | Not applicable – open-label |
| Methods – Statistical methods | 12a. Statistical methods for primary and secondary outcomes | Methods – Statistical analyses |
| Methods – Statistical methods | 12b. Additional analyses (subgroup, adjusted) | Methods – exploratory analyses, correlations |
| Results – Participant flow | 13a. Numbers randomized, treated, analyzed | Results – 56 patients included; arms A–D: 16,16,12,12 |
| Results – Participant flow | 13b. Losses and exclusions after randomisation, with reasons | Results - 5 patients discontinued RLT due to grade 3 thrombocytopenia |
| Results – Recruitment | 14a. Recruitment and follow-up dates | Methods – recruitment started 2022, interim cut-off after 56 patients completed RLT |
| Results – Recruitment | 14b. Why the trial ended or was stopped | Ongoing trial – not stopped |
| Results – Baseline data | 15. Baseline demographic and clinical characteristics | Results – Table 1 |
| Results – Numbers analyzed | 16. Numbers analyzed per group | Results – arms A–D, N per arm |
| Results – Outcomes and estimation | 17a. Outcomes, effect size, precision | Results – organ doses, hematology, renal/hepatic function; exploratory statistics |
| Results – Outcomes and estimation | 17b. Absolute and relative effect sizes for binary outcomes | Not applicable – safety/dosimetry only |
| Results – Ancillary analyses | 18. Other analyses, subgroup | Results – dose–effect relationships, correlation plots |
| Results – Harms | 19. Harms/unintended effects | Results – hematologic declines, renal stability, no hepatotoxicity |
| Discussion | 20. Limitations, bias, imprecision | Discussion – Limitations and Strengths |
| Discussion | 21. Generalisability of findings | Discussion – multicenter design, relevance for RLT practice |
| Discussion | 22. Interpretation consistent with results, benefits and harms | Discussion and Conclusions |
| Other information | 23. Registration number and trial registry | Methods - EudraCT 2020-006068-99 |
| Other information | 24. Protocol access | Not stated – available from funding agency/protocol |
| Other information | 25. Sources of funding and role of funders | Methods – funded by Medical Research Agency, Poland, project no. 2019/ABM/01/00077 |
